# Characterisation of the Utrerana Chicken Breed Farms in Spain

**DOI:** 10.3390/ani14243608

**Published:** 2024-12-14

**Authors:** Antonio Plata-Casado, Carmelo García-Romero, Pedro González-Redondo

**Affiliations:** 1 Consultancy in Organic Agriculture and Holistic Management, 41710 Utrera, Spain; asusl@hotmail.com; 2 Real Academia de Ciencias Veterinarias, Instituto de España, 28006 Madrid, Spain; guindalejocarmelo@gmail.com; 3 Departamento de Agronomía, Universidad de Sevilla, Carretera de Utrera Km 1, 41013 Seville, Spain

**Keywords:** zoogenetic resources, laying hen, self-supply farming, Andalusian countryside, herd-book, marketing, farm structure, local breed, poultry

## Abstract

With the aim to characterise the situation of the subsector, 25 poultry farms of the endangered native Utrerana chicken egg-laying-oriented breed (*Gallus gallus* Linnaeus, 1758) were surveyed in Andalusia (southern Spain) from 2021 to 2023 to investigate the structure of the farms, number of birds, health status, feeding management, and marketing of their products. It was found that the pace of foundation of Utrerana chicken farms accelerated from 2009, and most of the farms were concentrated in the province of Seville. Only 40% of the farms were legally registered. This breed is raised on small farms mainly dedicated to self-supply and raising birds for conformation and their aesthetic values, with limited marketing of eggs and meat, with facilities consisting often of one poultry house and one outdoor enclosure, and with a low number of laying hens per farm (53.5 on average). Current productivity is low, with an average of 139 eggs per hen per year, but hens have a long reproductive lifespan, averaging 4 years. The Partridge variety of the breed was the most common, followed by the Black-barred and Black varieties, while the White variety was very scarce, with only 31 specimens in total. Two-thirds of the farmers also raised other breeds of chickens, in addition to the Utrerana. The management and production system was free-range or backyard, with a certain proximity to the requirements of European Union-certified organic production with regard to stocking densities and generally with a good sanitary status, respect for welfare, and implementation of biosecurity measures on the farms. The feeding of the chickens was based on grazing in outdoor enclosures and on the provision of grains and compound feed, with 40.0% of farmers using commercial compound feed and half preparing feed themselves. Two-thirds of the farmers were affiliated with the National Association of Utrerana Chicken Breeders (“Asociación Nacional de Criadores de Gallinas Utreranas”, ANCGU, in Spanish); almost half of them took part with their birds in fairs and conformation shows, such as the Utrerana Chicken Fair held in Utrera (province of Seville) and more than a quarter of them have sold live birds of the breed at these fairs. The farmers carry out few promotional and advertising activities for their farms and products, predominantly by using social networks, and the market area reached is mainly local. In conclusion, this research has shown that the current alternative production system for Utrerana chicken is similar to that of the hens that originated the breed and consists of a few amateur farms, with a small number of birds, distributed mainly throughout the Andalusian countryside. It has been highlighted that there is a need for the livestock authorities to implement programmes for supporting farmers and promoting the breeding of this endangered avian breed, native to the Spanish zoogenetic heritage.

## Simple Summary

The Utrerana is an endangered egg-producing chicken breed native to Spain, whose commercial farming was abandoned in the second half of the 20th century, being currently raised by amateur farmers for self-supply and for the morphological beauty of the birds. The current situation and structure of the farms that maintain this breed has received little attention in terms of research. To address this gap, we characterised the Spanish Utrerana chicken farms subsector by administering a survey to farmers. We found that the Utrerana chicken farms were concentrated in the Andalusian region (southern Spain), their pace of foundation accelerated by the end of the 2000s decade, and they have a low number of laying hens (about fifty on average) raised under a free-range system in facilities consisting often of one poultry house and one outdoor enclosure. Egg yield per hen per year is low because genetic selection for productivity was abandoned decades ago. Feeding of the Utrerana chicken is based on grazing in outdoor enclosures and on the provision of grains and compound feed, and stocking densities, sanitary status, respect for welfare, and implementation of biosecurity measures on the farms are good. Marketing is essentially limited to the local area and to the sale of live birds and some eggs, partly because the farms are oriented towards self-consumption. Support from the livestock authorities is necessary to expand the breeding of this endangered breed and to promote the marketing of its products.

## 1. Introduction

The Utrerana is a Mediterranean-type chicken breed native to southern Spain, included in the Domestic Animal Diversity Information System (DAD-IS) of the Food and Agriculture Organization (FAO) [1] and in the Official Catalogue of Livestock Breeds of Spain [2,3], being endangered with a vulnerable local risk status. This avian breed was created in the municipality of Utrera (province of Seville, Andalusia) in 1926 at the Santa Matilde Farm by the poultry farmer Mr. Joaquín del Castillo [4,5,6]. The foundation of the breed started from white-shelled eggs weighing 80 g on average gathered in farmhouses and ranches in the Seville countryside, which were subsequently incubated, producing nearly 400 chicks, from which 200 hens and several roosters of varied feather colours were chosen to serve as breeding birds [5]. The main virtues of these animals were their notable body size and aptitude for laying large eggs [5,7], loss of broodiness [5], and rusticity and resistance to diseases [4,8,9]. Utrerana hen eggs are of good size, with an average weight of 62–64 g (shape index: 71.3–75.6%) and a white or off-white shell colour [5,7,10]. It is composed of 12.5–13.8% eggshell, 52.4–56.6% albumen, and 28.4–32.3% yolk [7].

The Utrerana chicken breed soon became famous and widespread and participated in various events of the time, obtaining some awards and official recognition as a breed wherever it participated [5,9]. Thus, in the egg-laying contests at which the Utrerana hens participated between 1939 and 1948, the records reached were 177–239 eggs/year, depending on the variety [5].

**Figure 1:**
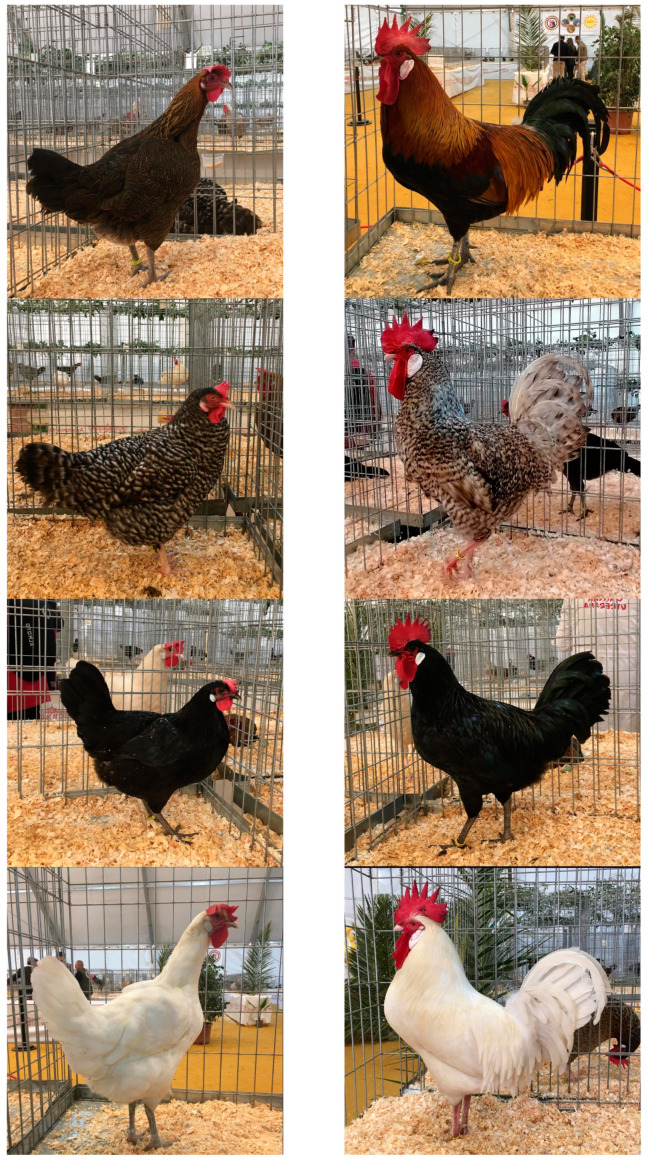
Varieties of the Utrerana chicken. From top to bottom: Partridge, Black-barred, Black and White. Hens (**left**) and roosters (**right**) (Pictures’ authors: Alicia Sanz and Pedro González-Redondo).

This breed has four plumage varieties: White, Black-barred, Black, and Partridge (Figure 1), defined by the colour of the feathers and legs, whose selection started in 1931 at Santa Matilde Farm by means of lines selection [5]. Initially, the Black, White, and Black-barred varieties were selected and fixed, and later the Partridge variety was extracted from the Black [11]. The Black-barred variety is also known as “Franciscana”, probably because Mr. Joaquín del Castillo named it that way because the Franciscan monks of the Utrera convent called “San Francisco el Viejo” raised chickens with this colour of feather between the 16th and 17th centuries [12]. The breed standards of the varieties of the Utrerana chicken achieved their first recognition in various Spanish poultry shows: the White in 1946, the Black-barred in 1948, and the Black and Partridge varieties in 1949 [5,9].

Genetic improvement and selection of the Utrerana breed reached such a level that, in the 1950s, hens of the breed achieved average annual laying of 180 large eggs (62–64 g on average) [5]. This allowed the Utrerana breed to reach a wide and rapid spreading and to be used in commercial poultry farming at the time [9,11,13,14,15], at the same level as other productive breeds of laying hens such as the Black Castellana (the most widespread and productive Spanish local breed [16,17]) and White Leghorn (a foreign breed that was already spreading in Spain, dominating thereafter intensive laying poultry farming [18]). In fact, the Utrerana chicken was raised in large groups in mainland and insular Spain (Canary and Balearic Islands), in Morocco, and in the Gulf of Guinea [5,9,11,14], being classified among the national productive or modern breeds [15].

However, from the 1960s onwards, with the widespread use of new, highly productive select strains of laying hens and the creation of intensive commercial laying farms nationwide with large numbers of hens and more economically profitable, conventional production on the ground with which the Utrerana breed had been farmed became unsustainable [15,18,19,20]. In the area around the breed’s origin, this was the case of the Santa Matilde farm and other farms in Utrera, such as El Campillo, which also joined in the production of Utrerana eggs in the second quarter of the 20th century [21]. Due to this economic crisis in the sector and the disappearance of these poultry farms, Utrerana hens were relegated from intensive commercial production and dispersed mainly throughout the Andalusian countryside [21]. This brought the breed to the brink of extinction. Orozco and Campo [22] verified, by the beginning of the 1980s, the existence of birds in various Andalusian farmhouses, which, being mixed, presented in segregation the colours of the Utrerana chickens. Cárceles [15] reported that the Utrerana breed could still be found in very few numbers in the Andalusian countryside. Rodero et al. [23] reported having found at the beginning of the 1990s hens that derived from the Utrerana breed but crossed with foreign breeds. Lancho [20] estimated the census of the breed in 1998 at about 800 birds. The populations of the White variety were the most reduced in this period due to the disappearance of commercial poultry farms for egg production, in which it was widely used [6,14].

In recent times, starting in the mid-1970s, very few poultry farmers with small farms managed to prevent the total extinction of the Utrerana chicken, with Mr. Juan Manuel Sánchez Ocaña, Mr. Antonio Vélez Marchena, Mr. Ángel Martínez Muñoz, and Mrs. Encarnación García Román being the main supporters and retainers of this breed at that time [4,21,24]. Later, after an initial failed attempt, the official recognition of the Utrerana breed to be included in the Official Catalogue of Livestock breeds in Spain, with the category of autochthonous breed, was approved at the meeting of the Committee of Livestock Breeds of Spain on 21 June 2006, thanks to the efforts of the Asociación Andaluza de Avicultura (Andalusian Poultry Association) and the technical work of the Agricultural Provincial Center of the Provincial Council of Cordoba [6,25,26]. In 2009, the Asociación Nacional de Criadores de Gallinas Utreranas (ANCGU; National Association of Utrerana Chicken Breeders) was created, initially integrating several poultry farmers from the Seville countryside area [27], and in December 2013 the ANCGU was granted, by the General Directorate of Agricultural and Livestock Production of the Regional Government of Andalusia, the right to keep the Herd-Book of the breed [28,29]. These milestones began a new period of growth and expansion of the breed at a national level. Currently, there are 42 poultry farms that maintain the 1339 birds registered in the Herd-Book [3,30].

As a consequence of this historical development, the Utrerana chicken farms that have survived to the present day are small in size, having greatly reduced the number of birds per farm. These are maintained by amateur poultry farmers who keep the birds as a hobby, destined for self-supply and for breeding and selecting birds for morphological traits and aesthetic values, for participation as exhibition animals in fairs and conformation shows, with these farms being widely spread, mainly in the Andalusian countryside [6,24]. Due to this particularity of being small farms, with an outdoor enclosure or pen and natural feeding and with a unique, traditional management, they could potentially obtain the certification of organic production [4,6].

In this context, the productive and functional [7], as well as genetic [31], characterisation of the breed, have been carried out (extensively reviewed in Plata-Casado et al. [6]), but there is a lack of knowledge about the current situation of Utrerana breed poultry farms and the breeders who maintain it, which points to the need to undertake a systematic characterisation of this subsector. The characterisation of livestock farms has been widely used in research into animal production systems, both in major livestock species [32,33,34] and in alternative livestock farming [35,36,37,38], as well as in chicken farming [39,40]. This methodology for analysing animal production systems has been confirmed as a useful tool to expand knowledge about livestock subsectors and to help the competent authorities implement and manage farm support programmes, as well as to help technicians and veterinarians in technical-economic advice to livestock farmers [33]. In fact, in the context of a strategy formulated by the ANCGU, the Utrera City Council, Fundación Savia, and other agents for the valorisation of the Utrerana chicken and its products [41], one of the measures that has been proposed is the characterisation of the farms that raise this local breed [6] to get reliable and first-hand information that will contribute to its conservation and expansion.

Therefore, the main objectives of this study are to analyse and understand the current situation of farmers of the Utrerana chicken breed and to characterise the farms where it is raised in its main distribution area in Spain, using variables related to the structure, facilities, marketing and advertising, as well as productive, feeding, and sanitary management. This will provide relevant knowledge on these small farm systems and their diversity, useful in helping to the conservation of this endangered local zoogenetic resource.

## 2. Materials and Methods

### 2.1. Study Area and Sample Searching

The study was conducted in Andalusia (Spain), as it is the most relevant region where the Utrerana chicken is raised [6]. Searching for farms and inviting them to take part in the survey was carried out in several ways, mainly by contacting members of the ANCGU, as well as by personal contacts with other non-member farmers. All the Utrerana chicken farmers found were contacted and invited to participate in the study. The sample gathered consisted of 25 farms located in five provinces whose distribution was representative of the current geographic location of the Utrerana chicken farms in Andalusia [6]. The sample amounted to 59.5% of the 42 farms registered in the Herd-Book [3] when the survey was conducted.

### 2.2. Data Collection and Variables Studied

**Figure 2:**
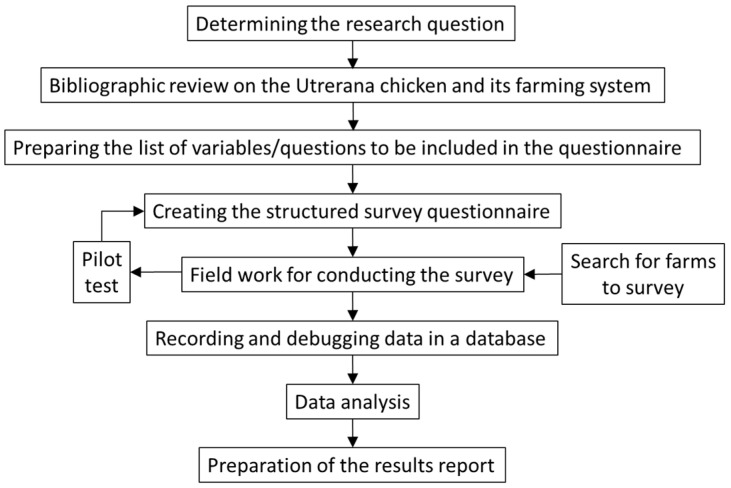
Flow chart of the survey process.

The information was gathered by a survey whose flow chart is shown in Figure 2, and whose fieldwork was carried out between 2021 and 2023 by directly interviewing the farmers at their farms, who participated voluntarily and with informed consent. The structured questionnaire included 53 dichotomous variables (“Yes” or “No” answers) and 18 quantitative variables, as well as ten open-ended variables, belonging to the thematic groups shown in Table 1. These variables were chosen on the basis of a review of previous knowledge on the Utrerana chicken breed and its raising [6].

### 2.3. Statistical Analysis

The mean, standard error of the mean, mode, minimum, and maximum were calculated for quantitative variables, such as the number of birds by variety and sex, number and surface area of poultry houses and outdoor enclosures or pens, temporal frequency of rotation of birds among outdoor enclosures or pens, annual egg production per hen and per farm, reproductive lifespan, culling rate, and temporal frequency of litter removal from the poultry houses. For the variables that had several modes, the lowest modal value was chosen.

For the other variables of the survey, which were dichotomous, the number and percentage of farms showing each attribute were calculated.

The statistical analyses were performed using IBM SPSS Statistics for Windows v. 29.0.2.0 software (IBM, Armonk, NY, USA).

## 3. Results

This section presents the systematic characterisation of the Utrerana chicken breed farms surveyed in this study, carried out by using variables related to animals, facility structure, management, production, as well as promotional and marketing activities.

### 3.1. Geographical Location and Year of Establishment of the Farms

**Figure 3:**
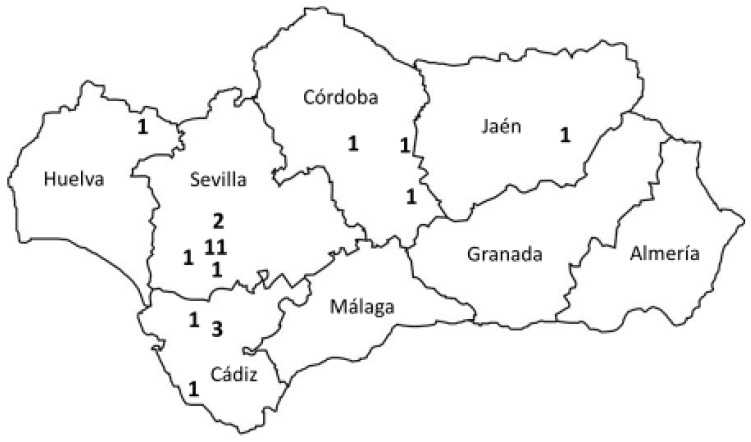
Geographical location map in Andalusia of the Utrerana chicken farms surveyed (n = 25). Each figure represents the number of farms surveyed in a municipality located where the figure is typed.

All poultry farms surveyed in this study were located in the Autonomous Community of Andalusia (southern Spain; Figure 3), with Seville, with 15 farms (60%), being the province with the highest concentration of poultry farmers raising the Utrerana chicken breed. Other provinces where there were farmers of this breed interviewed were Cadiz, Cordoba, Huelva, and Jaen.

**Figure 4:**
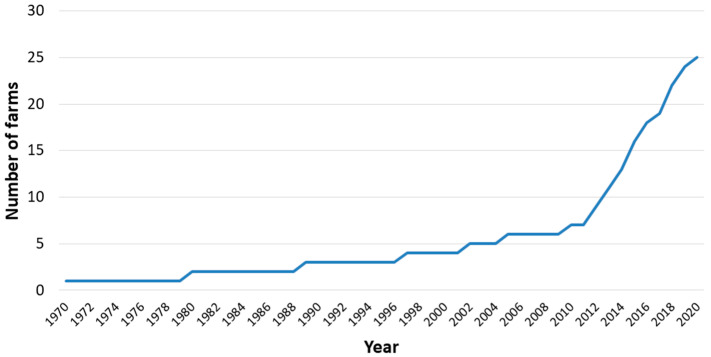
Number of Utrerana chicken farms surveyed according to the year of establishment (n = 25).

The oldest Utrerana chicken farm sampled was created in 1970, with farms being subsequently founded at a slow pace until 2009, when the start-up of new farms of the breed accelerated (Figure 4).

### 3.2. Bird Census and Farm Size

**Figure 5:**
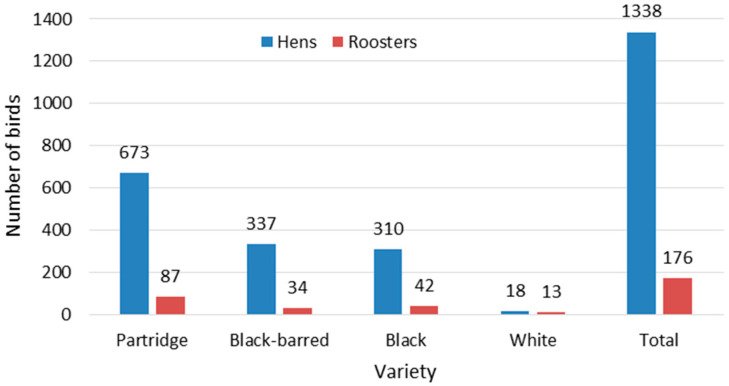
Total aggregated census of Utrerana breed hens and roosters, by varieties, in the surveyed farms (n = 25).

The total aggregate number of birds of all varieties of the Utrerana chicken breed and both sexes present on the surveyed farms was 1514 (Figure 5). There were notable differences in the numerical consistency of the populations of the different varieties of the breed, with the Partridge variety being more abundant, with half of the census (637 hens and 87 roosters). It was followed by the Black-barred (337 hens and 34 roosters) and Black (310 hens and 42 roosters) varieties, with almost one-quarter of the census each. The White variety (18 hens and 13 roosters) was scarcely represented, with only 2% of the total census, although with a higher proportion of roosters per number of hens compared to the other varieties (72.2% vs. 10.1–13.5%).

Table 2 shows the proportion of farmers who kept birds of the different varieties of the Utrerana chicken breed, as well as the number of birds of each variety by sex. The most widespread variety was the Partridge, with 84% of farmers keeping an average of 32.1 hens and 72% having 4.83 roosters. It was followed by the Black-barred variety, with 60% of farmers having an average of 22.5 hens and 40% keeping 3.40 roosters, as well as the Black variety, with 56% of farmers having an average of 22.1 hens and 52% keeping 3.23 roosters. The White variety was the least widespread, with 20% of farmers having an average of 3.60 hens and 24% having 2.17 roosters. The average farm size was 60.6 birds (53.5 hens and 7.33 roosters), although it was highly variable.

### 3.3. Administrative Status and Productive Specialisation

Table 3 shows the administrative registration modalities of the Utrerana chicken farms, their productive specialisations, and the production of other types of livestock.

The administrative status of the Utrerana chicken farms is characterised by the fact that only 40% were included in the General Registry of Livestock Farms (“Registro General de Explotaciones ganaderas”, REGA, in Spanish) and none of them was registered as a Zoological Nucleus. Only one farm was certified for organic egg production. The main reasons why respondents have not certified their farms for organic production are that in some cases they are unaware of this option, and in other cases they are not interested.

Most of Utrerana breed chicken farms (72%) were self-supply or hobby farms, while 16 and 72% produced meat and eggs for sale, respectively. Fifty-six percent of the surveyed farms produced breeder birds to be sold.

In addition to Utrerana breed chickens, two-thirds of the surveyed farms also raised other breeds of chickens, such as Andaluza Azul, Moñuda Andaluza, Sureña, Combatiente Español, Leghorn or Isa Brown, and 44% raised other livestock species, such as horses, sheep, goats, cows, pigs, rabbits, pigeons, turkeys, mallards (*Anas platyrhynchos*), red-legged partridges (*Alectoris rufa*), or greylag geese (*Anser anser*).

Half of the poultry farmers interviewed grew agricultural products on their farms, such as olive trees, vegetables, cereals, or orange trees.

### 3.4. Farm Facilities and Equipment

The Utrerana chicken farms had an average of 2.92 poultry houses, although more frequently they had only one, and 12 at most, with an average surface area of 179 m^2^, ranging between 6 and 2000 m^2^, and most frequently 30 m^2^ (Table 4). In 96% of the farms (n = 24) the chickens had access to outdoor enclosures or pens (Table 4), while in one of them (4%), they were permanently housed in an indoor closed poultry house. They had an average of 1.72 outdoor enclosures or pens where the chickens could go out to graze and sunbathe, varying between a most frequent value of one and a maximum of 9, which occupied 3381 m^2^ on average, varying between a most frequent area of 1000 m^2^ and a maximum of 12,000 m^2^ (Table 4).

Forty-eight percent (n = 12) of the Utrerana chicken farms rotated the birds among outdoor enclosures or pens, with a frequency of every 22.4 ± 3.66 weeks, varying between 1 and 36 weeks and most often 24 weeks.

Table 5 shows the frequency of Utrerana chicken farms that met the minimum surfaces for indoor and outdoor areas laid down by the Commission Implementing Regulation (EU) 2020/464 [42] for the stocking densities ensuring a high level of animal welfare in the certified organic farming. The maximum density of birds inside the poultry houses did not exceed the values laid down in said regulation for laying hens in 68% of the surveyed farms and for fattening chickens in 60% of them. Conversely, the minimum surface area of 4 m^2^/bird in the outdoor areas, laid down by the said regulation, was guaranteed in 92% of the surveyed farms.

Table 5 also shows the frequency of Utrerana chicken farms that met the minimum space available per bird in each kind of feeder and drinking trough, laid down by the Royal Decree 3/2002 [43] to ensure animal welfare in laying hens. The minimum space per bird was sufficient in 56% of farms with linear feeders and in 76% of farms with hopper feeders. Only 12% of the farms had automatic feeders. In the same vein, the minimum space per bird was sufficient in 52% of farms with channel drinking troughs, in 28% of the farms with nipple or cup drinking troughs, and in 20% of farms with circular drinking troughs. Half of the farms had automatic drinking troughs.

### 3.5. Egg Production and Reproductive Lifespan

Average egg production per hen per year in the Utrerana chicken breed farms surveyed was 139, with a maximum of 210 and a more frequent production of 100 eggs per hen per year (Table 6). Total annual egg production per farm averaged almost 5000 eggs, reaching a maximum of 40,800, with 2000 eggs being the most frequent production.

The lifespan of Utrerana breed chickens lasted, on average, 49 months on the farms surveyed, reaching a maximum of 8 years but most frequently lasting 5 years. The farmers stated that they applied an average culling rate of 15.4%, although it varied between a more frequent value of 10% and a maximum of 60%.

### 3.6. Husbandry Practices

Table 7 shows several husbandry and management practices carried out in the Utrerana chicken breed farms surveyed.

One-third of Utrerana poultry farmers composted bird manure to obtain fertiliser for their crops. Regarding reproductive husbandry, only one farmer carried out artificial photoperiod supplementation to stimulate hens’ egg-laying capacity. Sixty-eight percent of poultry farmers incubated fertile eggs from their hens; 40% of the total used an artificial incubator, and 48% resorted to natural incubation with hens.

Two-thirds of poultry farmers slaughtered fattening chickens with at least 81 days of age. Most of the roosters and some of the hens that are not intended for laying eggs are slaughtered, at average body weights of 3 and 2.3 kg, respectively. In this regard, 80% of farmers considered that there is a variation in the quality of the meat of Utrerana chickens compared to other breeds, characterised by its more intense colour, flavour, firmness, and fibrous texture.

### 3.7. Feeding Management

Table 8 shows the main practices related to the feeding management of Utrerana chickens carried out by the poultry farmers surveyed.

Sixty percent of farmers grew vegetables specifically destined to feed their birds, such as chard, lettuce, beans, peas, tomatoes, oat, barley, brassicas, sow thistle, or alfalfa. In addition, in half of the farms the birds had access to plots with trees.

Forty percent of the farmers used commercial (corn-based) balanced compound feed to feed their birds, and only one farmer was aware of whether the compound feed included raw materials containing genetically modified organisms. Almost half of the poultry farmers prepared their own feeds for their birds, using mainly wheat, maize, rice chaff, sunflower seed, sorghum, barley, peas, and broad beans; and 40% gave them vitamins and minerals.

### 3.8. Biosecurity, Animal Welfare and Pathology

Table 9 shows biosecurity and prophylaxis measurements implemented by the Utrerana chicken farmers interviewed in the study. Ventilation of poultry houses was considered adequate in all cases except one. In the same vein, all but one poultry farmer took protective measures against rodents, disinfected the feeders and drinking troughs, and removed the dead birds. Eighty-eight percent of them removed the litter from the poultry houses, with a frequency of 9.82 ± 1.97 weeks, varying between 1 and 36 weeks, and most frequently every four weeks. Only 28% of poultry farmers vaccinated their birds, mainly against fowl pox, infectious coryza, virulent Newcastle disease, and infectious bronchitis, as well as against infectious bursal disease and Marek’s disease in one of these farms.

Twelve percent of poultry farmers reported an incidence of intestinal parasites, 24% reported an incidence of mites, and one-fifth reported that their birds had suffered from respiratory diseases. No one reported diarrhoea (Table 9).

None of the poultry farmers trimmed the birds’ beaks or plucked them alive (Table 9).

One poultry farmer belonged to a Livestock Health Defence Group.

### 3.9. Marketing, Promotion and Advertising

Table 10 shows the marketing, promotion, and advertising activities carried out by the Utrerana chicken farmers. Two-thirds of the farmers were affiliated with the ANCGU, while two farmers belonged to another association (“Asociación Avícola Barqueña”, based in La Barca de la Florida, province of Cadiz). Forty-four percent of the poultry farmers participated with their birds in fairs and conformation shows, and 28% have sold birds at these events. One-third of the farmers surveyed have received prizes at these fairs and conformation shows for the Utrerana chickens presented at them. Only one farmer received subsidies for raising this breed.

Regarding the promotional and advertising activities of the Utrerana chicken carried out by the farmers, only 8% of them had their own website, and a quarter of them used social networks to promote their birds and products. Only one farm has advertised in the press, and one farmer has published magazine articles about the Utrerana chicken breed.

Forty-four percent of the interviewed farmers have sold hatching eggs; almost three-quarters have sold table eggs, and a sixth sold Utrerana chicken meat.

Forty percent of the Utrerana breed farmers reached only the local market, 8% reached the regional market of the Autonomous Community of Andalusia, and only one of them had sold their birds on a nationwide scale. None of them had exported Utrerana chickens.

## 4. Discussion

Throughout the almost century-long existence of the breed, informative and scientific literature on the Utrerana chicken has addressed aspects such as its reproductive [44,45], productive [7,46,47], genetic [31], functional [7], and product quality [10,48] characterisation. Moreover, Plata-Casado et al. [6] recently undertook a critical review, comprehensively analysing the state of the art of this avian breed. However, as far as our knowledge goes, the present research constitutes the first systematic characterisation of farms that raise Utrerana chickens in their main distribution area, carried out by directly interviewing farmers and by recording and analysing variables related to bird census, structure of facilities, husbandry and management, sanitary status, marketing, and promotional activities. The results obtained through this characterisation provide a detailed overview of the current situation of Utrerana breed poultry farms and the management carried out by farmers on their respective farms. This knowledge, discussed below, is useful for generating proposals aimed at the conservation, valorisation, and promotion of the breed, as well as for the authorities responsible for livestock farming and conservation of local breeds to be able to implement support programmes for farmers and the Utrerana chicken breed.

According to the Official Catalogue of Livestock Breeds of Spain [3], there are 40 Utrerana chicken farms registered in the Herd-Book, although there are actually 42 farms because two of them integrate two independent facilities with the same official registration number (REGA). In addition, there is an undetermined number of farms that keep Utrerana chickens, which are not registered in the Herd-Book. Thus, the 25 farms surveyed in the present study represent almost two-thirds of the number of farms that are registered in the Herd-Book. Although outside Andalusia the Herd-Book includes two registered farms located in the regions of Castilla-La Mancha and Extremadura [3], all the farms participating in this study were located in Andalusia, with 60% of them located in the province of Seville and 44% in the municipality where the breed was founded and which gives its name to it, Utrera [5] (Figure 3). This means that, currently, the farms that maintain the Utrerana chicken have practically remained located in the area of origin of the breed, having lost the widespread national and even international diffusion it reached at its heyday in the 1950s, when it was widely used in commercial egg production in mainland Spain, the Balearic and Canary Islands, the Gulf of Guinea, and Morocco, among other territories [5,9,11,14].

This drastic reduction in the Utrerana chicken farms’ distribution area was due to the abandonment of their commercial farming due to the crisis in the poultry sector, which occurred by shifting from eminently family-based, self-supply, and small-scale production models in chicken houses on the ground to industrial production based on American technology and genetics, which accelerated very intensely in Spain in the 1960s [49]. In fact, during the next three decades, the literature attests to the virtual disappearance of farms of the Utrerana breed, while at the same time in Andalusia there was a reduction to one-eighth of the number of farms of all poultry production systems and breeds, increasing the number of birds per farm [50]. Thus, Orozco and Campo [22] and Cárceles [15] by the beginning of the 1980s, and Rodero et al. [23] at the beginning of the 1990s reported that the Utrerana chicken could still be found in very few numbers in the Andalusian countryside and often crossed with foreign breeds. This explains why the establishment of the farms surveyed in this research did not begin until 1970 and the pace of their foundation did not accelerate until the end of the 2000s (Figure 4). This recent resurgence of farms that raise the Utrerana breed was due to a series of milestones, among which are the selfless work of poultry farmers such as Mr. Juan Manuel Sánchez Ocaña [4,6,24], and others mentioned in the Results section, and research centres such as the Provincial Agricultural Centre of the Provincial Council of Cordoba [26,51,52], to preserve the breed, the inclusion of the breed in the Official Catalogue of Livestock Breeds of Spain in 2007 [25], the creation in 2009 of the ANCGU [27], and the concession in 2013 to this association for the maintenance of the Herd-Book [28,29].

A similar dynamic to that of the geographical distribution and number of Utrerana chicken farms has been followed by the census of birds of the breed. In fact, at the beginning of the 1950s, Piedrafita [9] estimates that a million and a half Utrerana chicks were produced per year, and Liaño et al. [11] report that the breed was usually bred in large flocks in Spain, because it was broadly used as a productive laying hen [13]. However, our study revealed a drastic reduction in the current numerical consistency of the Utrerana chicken population, totalling just over 1500 birds of both sexes and four feather varieties across all the farms surveyed (Figure 5). It is difficult to reliably estimate the numerical abundance of the breed’s population due to the absence of specific censuses, but considering that in the present study we have surveyed a number of farms equivalent to 60% of those registered in the Herd-Book, the total census of the breed could be estimated at about 2500 birds. These estimates are in line with the total number of birds of the Utrerana chicken breed registered in the Herd-Book being 1339 and would confirm the recent trend towards its more recent expansion [3], particularly when compared to the 800 birds estimated by Lancho [20] in the late 1990s.

There is no previous statistical information to establish evolutionary comparisons of the subsector, but currently the farms that keep Utrerana chickens are very small, with an average of 60.6 birds with 88% hens and 12% roosters (Table 2), having become oriented towards self-supply and bird keeping as a hobby [6]. The number of birds of each feather variety of the breed kept by farmers was quite different (Table 2), with the Partridge variety predominating, which was kept by three quarters of the poultry farmers surveyed (keeping an average of 32.1 hens and 4.83 roosters), followed by the Black-barred (22.5 hens and 3.40 roosters) and Black (22.1 hens and 3.23 roosters) varieties kept by just over half of the farmers, and the White variety, which was kept only by a fifth of the poultry farmers (with an average of 3.60 hens and 2.17 roosters). The White variety was the most affected by the crisis in the sector described above, almost disappearing and remaining with a census of 13 roosters and 18 hens in all the farms surveyed (Figure 5). The relative abundance of each variety evidenced in this study, which was 50.2, 24.5, 23.3, and 2.00% of birds of the Partridge, Black-barred, Black, and White varieties, respectively, reveals a relative increase in birds of the Partridge and Black-barred varieties and a decrease in birds of the White variety with respect to birds registered in the Herd-Book in 2022 [29], which was 35.0, 10.60, 32.4, and 8.50%, respectively, as reported in Plata-Casado et al. [6]. The greater abundance of birds of the Partridge, Black, and Black-barred varieties and the residual presence of the White variety has also been observed in the conformation shows of the Utrerana Chicken Fair held during the last two decades [6]. The current predominance of the Partridge variety contrasts with the fact that it was the last of the four varieties to be selected and fixed [11] and, in fact, in 1950 its process of selection was not yet completed [9]. Rabanal [14] reports that the White, Black, and Black-barred varieties of the Utrerana breed were among the most used laying hens in 1948, while he does not mention the Partridge variety.

In Spain, livestock farms must be registered in the General Registry of Livestock Farms (REGA) in order to keep animals and market their products [53]. The relatively low percentage of Utrerana chicken farms that are registered in the REGA (40%) is related to the fact that the majority of them (72%) are hobby and self-supply farms that are not oriented towards the commercialisation of products, an aspect that is discussed later but which is confirmed because only 16% of the farms produce meat and 72% produce eggs (Table 3). It is surprising that none of the Utrerana chicken farms was registered as a zoological nucleus, an alternative legal registration modality for farms aimed at the registration and zoosanitary authorisation of private zoological and pet animal collections suitable for the keeping of animals of different species for hobby and self-supply [54]. In fact, two-thirds of the poultry farmers surveyed also kept other breeds of chickens, and almost half of them raised other livestock species (Table 3). It is therefore necessary for the livestock authorities and the ANCGU to encourage farmers of Utrerana chickens to legally register their farms, as required by the Royal Decree 637/2021 for the regulation of poultry farming in Spain, either as self-supply, which would permit 30 laying hens and 50 fattening chickens under the condition of not selling products, or as a reduced farm, which would also allow them to market products for human consumption [55,56].

The current breeding system of the Utrerana chicken is very similar to that of the animals of the breed in their origins, when birds were reared under a free-range regime with feeding based almost exclusively on the natural resources of the country with little supplementation of grain [4,5], as confirmed by the fact that all but one of the farms surveyed had outdoor enclosures or pens (Table 4) with herbaceous vegetation and trees for shelter, where the chickens feed, graze, and live outdoors. In line with their small flock size (Table 2) and their self-supply orientation (Table 3), the number of poultry houses and outdoor enclosures per farm was small, often having only one facility consisting of a poultry house with its connected outdoor enclosure. However, these facilities were spacious (Table 4), which would allow a majority of Utrerana poultry farmers to meet the legal requirements of maximum bird density in the poultry house (two-thirds of the farms) and minimum space per bird in outdoor enclosures (almost all farms) (Table 5) established by the European Union organic production regulations [57], in particular the Commission Implementing Regulation (EU) 2020/464 [42], since poultry farmers who have several enclosures rotate them quite frequently. However, despite this proximity to the characteristics of the organic production system, due to a number of reasons described in the Results section, only one poultry farmer is certified for organic production. Most farmers, due to lack of knowledge and others for not having this certification, as well as due to administrative obstacles, continue working with this breed in self-supply modality. In this regard, it would be interesting to undertake measures to support the conversion of Utrerana chicken farms to this certified production system, as has been advocated on various occasions [4,6,56], even more so taking into account that the Utrerana breed adapts to these alternative production systems [4,8,58]. In fact, two-thirds of the farmers slaughtered fattening chickens at an age of at least 81 days (Table 7), which is the minimum age required for certified organic production [57], showing in this respect a relatively good proximity to the certified organic production system.

As expected in small-scale, non-professional farms [59], the level of automation in feed distribution was very low among the farms surveyed, with only one-eighth of them having automatic feeders, while half of the farms have automatic drinking troughs (Table 5). This is explained by the high cost of automating this equipment, which only pays off on farms of a certain size [60]. On the other hand, the provision of feeders and drinkers per number of birds in the Utrerana chicken farms surveyed varied according to the type of feeders and drinkers used by the poultry farmers (Table 5), although, in general, it was moderately insufficient to meet the specifications of Royal Decree 3/2002 [43], which establishes the requirements that facilities must meet for the protection of laying hens in alternative production systems in Spain.

As indicated above, the Utrerana chicken breed is characterized by its egg-laying orientation [4,5], and, in fact, at the time of its maximum diffusion, it was used in commercial egg production together with other local Spanish and foreign breeds [14], which reached average productions of about 180 eggs per hen per year [5]. However, the average annual production per hen reported by the poultry farmers surveyed in the present study was lower, at 139 eggs, with more frequent values of only 100 eggs (Table 6). These values are close to the 111 eggs laid per hen and year in the founding stock before the selection of the breed began in 1926 [5] and are in the range of 94–121 eggs per hen and year, depending on the variety, reported in a recent investigation [46]. This drop in productivity of the breed was due to the abandonment of its productive farming and genetic selection after the emergence of the white and brown laying hen strains that have dominated intensive poultry farming in Spain since the second half of the 20th century [6,18], then becoming the Utrerana breed to be selected by conformation by the breeders who have maintained the breed during the last decades [6]. In fact, current Utrerana hens show seasonality in egg laying, with more or less prolonged periods without laying eggs in autumn and winter [10]. The low egg productivity of the Utrerana hen confirmed by the poultry farmers in the present study is also due to the fact that only one of them used artificial photoperiod supplementation to stimulate egg laying (Table 7), a practice that, on the contrary, is usual in egg production farms and allows egg-laying performance to be maximised [60,61,62]. As already proposed, it would be interesting to valorise the Utrerana breed for its use in alternative production systems, such as organic farming, by undertaking a programme to improve and select the breed for egg productivity [6,56]. In fact, the present research shows that current Utrerana hens have a wide variability in egg production, something necessary to be able to apply high selection pressure and achieve genetic progress in an eventual improvement programme [63], since their annual laying reaches up to 210 eggs per hen in some of the surveyed farms (Table 6).

The reproductive longevity of the Utrerana hen reported by the poultry farmers surveyed is very high, four years on average and five years on most farms (Table 6). This long reproductive lifespan coincides with the longevity of between 3 and 6 years previously described for this breed [4] and with that reported for other native breeds [64] and is notably greater than the lifespan of hens from select lines on intensive commercial farms, which does not usually extend beyond two years of age [61,62]. This is due to the breed’s rusticity [5], lower laying intensity [46], and seasonal rest [10], which imply less physiological deterioration for the hen. Consequently, the culling rate was low in the surveyed farms (Table 6).

The management practices carried out by Utrerana chicken farmers are similar to those commonly followed on farms of other breeds of egg-producing chickens with analogous free-range and backyard production systems [59]. Only one-third of farms composted their manure (Table 7), partly because only half of the farmers had agricultural crops (Table 3) on which to valorise the compost [65,66], and partly because on all but one farm the birds spent most of the day in the enclosures, with most of the droppings being incorporated directly into the soil [67]. Two-thirds of poultry farmers incubated eggs to breed Utrerana chickens, either using artificial incubators [60], natural incubation by hens [59], or even combining both systems (Table 7).

The results of the survey confirm that the feed management carried out by Utrerana chicken farmers corresponds to that typical of backyard farms [59,68] and is very similar to the traditional feed management of this breed in its origins, in which the basis of the diet consists of herbs, seeds, and bugs found by chickens in outdoor enclosures [4,8]. In fact, in all farms except one, the chickens had access to open-air enclosures, in half of the cases with trees for the birds to rest and protect (Table 8), and in almost two-thirds of the farms, vegetables were grown for the chickens’ feeding (Table 8). As is usual in this type of small farm, relatively few farmers use commercial compound feeds (Table 8) [69], which are available in an accessible supply in seed shops and livestock supply stores [70], while half of them prepare their feed from various raw materials and ingredients (Table 7) reported in the Results section. The fact that 40% of farmers supplemented their birds’ feed with vitamins and minerals (Table 8) indicates that the feeding practices followed by them were adequate [69].

This research has confirmed that the hygiene-sanitary and welfare status of the Utrerana chickens and their farms are adequate, even though the vaccination rate was low (Table 9), since the incidence of the main pathological disorders typical in chickens [71] was low or null (Table 9). This favourable situation is contributed to by the rustic character of the Utrerana breed [4,5,8], the low housing density, and the access of the birds to outdoor enclosures (Table 4 and Table 5), as well as the optimal level of compliance with the main biosecurity measures [72] in all the surveyed farms except one (ventilation of the henhouses, rodent control, periodic cleaning and disinfection of feeders and drinkers, periodic litter removal, etc.; Table 9), which are key to the fight against some diseases such as avian influenza or salmonellosis [71]. The proper welfare of the chickens in the farms surveyed was also confirmed by the fact that in no case were the birds’ beaks trimmed, a common practice in poultry farming to control pecking and reduce feed waste [73], since the integrity of the beak is necessary for the chicken to easily graze and feed on what it finds in outdoor enclosures [74]. In the same vein, no respondents plucked feathers from birds’ wings, a form of mutilation detrimental to the welfare of birds by impeding their natural flight behavior, prohibited by Regulation (EU) 2018/848 regulating organic production [57], that is common in free-range systems to prevent chickens from flying away from enclosures, particularly in native breeds that tend to be more agile and have some ability to fly [75]. In line with the fact that the farms surveyed were not professional businesses, only one poultry farmer belonged to a Livestock Health Defence Group, an association of owners or holders of animal farms legally constituted for the elevation of the sanitary and productive level and the improvement of the zootechnical conditions of their farms through the establishment and execution of prophylaxis programmes, the fight against animal diseases, and the improvement of their hygienic and productive conditions [76].

Two-thirds of the poultry farmers surveyed were members of the ANCGU (Table 10), which offers relevant services for the dissemination and promotion of the breed, as it actively participates in poultry fairs in Andalusia, such as the Loja Agricultural and Livestock Fair (“Real Feria de Ganado”, Loja, Granada province), Avicor (“Feria Avícola de Córdoba”, Cordoba province), and Fegasur (“Feria Nacional de Agricultura y Ganadería”, Jerez de la Frontera, Cadiz province) [77]. It also organises the Utrerana Chicken Fair (“Feria de la Gallina Utrerana”) in Utrera (Seville province), where the Spanish morphological competitions for this breed are held [6], live animals are sold, and commercial relations are fostered with other poultry farmers and the general public who are interested in the Utrerana chicken. In fact, almost half of the farmers surveyed had participated in these fairs and morphological competitions, with their birds having received prizes in a third of the cases and more than a quarter of the respondents having sold birds at these fairs (Table 10). To understand the relevance of these activities, it must not be forgotten that the Utrerana chicken is currently bred largely for its beauty and morphology, and not so much for the profitable production of eggs and meat [6].

Poultry farmers who kept Utrerana chickens made limited use of the various promotion and advertising channels available to promote their activity and products (Table 10). The proportion of them who maintained their own website (8%) is very low compared to game farms for red-legged partridge, pheasant, quail, or wild rabbit, which varies between 38.1 and 85.7% [35,36,37,38]. A quarter of them used social networks on their smartphones, mainly Facebook, Instagram, and WhatsApp, as promotion and communication channels. Given that the digitalisation and the use of the Internet and smartphones are widespread in today’s society, including in agricultural and livestock businesses [78,79], the limited use of these communication channels by Utrerana poultry farmers is striking. Advertising their products or birds in the press was irrelevant as a channel to promote them. Only one farmer had published magazine articles to divulgate the breed and its characteristics.

The marketing of Utrerana chicken products by the surveyed farmers is scarce (Table 10), partly because the farms do not have the mandatory legal registration and authorisations to do so, as they are limited-sized, self-supply farms that are not authorised to sell products [55], and partly because, to a large extent, the Utrerana breed is bred and maintained as a hobby due to its beauty as a pure breed [6] and not so much to obtain edible products. This explains why only one-sixth of the surveyed farmers sold Utrerana chicken meat, which may also be due to the added difficulty for such smallholder poultry farmers of having to carry out the slaughter of the birds in a slaughterhouse, the administrative obstacles to on-farm slaughter [80], or the lack of willingness of sellers and distributors to accept domestically produced chickens [81]. On the contrary, the marketing of eggs for human consumption was relevant, being carried out by almost three quarters of the farmers, most of them without specific authorisation to do so. On the other hand, another relevant part, 44% of the respondents, sold fertile eggs so that other interested persons can raise birds of the breed, a practice that is frequent in this type of poultry farm [82] and in other alternative subsectors, such as red-legged partridge, pheasant, or quail game farms, 19.0, 15.4, and 4.8% of which, respectively, sell fertile eggs [35,36,38].

Closely related to the limited marketing of products, and taking into account that the distribution area of the farms surveyed was limited to Andalusia and, in many of them, to the Seville province countryside, the geographical area of marketing was basically local, which was reached by 40% of the farms. To a lesser extent, marketing was achieved at a regional scale (8%) or throughout the whole Spanish territory (4%), while no farm carried out commercial operations at an international level. This poor market access is a common restriction in the marketing of products from local minority breeds and in the production of village chickens [40] and has also already been reported for the Utrerana chicken, having been proposed in this regard the need for its improvement through the valorisation of its products and other measures to support the subsector [56,83].

This characterisation of the farms that maintain the Utrerana chicken breed has illustrated the praiseworthy and altruistic work carried out by these farmers in favour of the conservation of the breed, as well as the need for the competent authorities and other agents to implement specific support programmes. In fact, only one of the poultry farmers surveyed received subsidies for maintaining the breed (Table 10). Some of the measures for the promotion of the Utrerana chicken breed and the dissemination of its breeding were proposed by Plata-Casado et al. [6], and several of them are being addressed by the ANCGU, also in the context of a strategy for the valorisation of the Utrerana chicken implemented by this association jointly with the Utrera City Council, Fundación Savia, and other agents [41]. In view of the results of this characterisation of the subsector, some of the measures that would be of interest to implement in support of farmers and the promotion of the Utrerana chicken breed would be: (i) recovering the hens with the highest egg production in the current flocks, through two initiatives: the organisation of a national laying contest to select those hens with the best laying performance, around 180 eggs per year, and the development by ANCGU of an advisory on-farm programme for farmers to improve laying performance of their hens; (ii) conduct research into the quality of Utrerana chicken meat, which has received little attention from the research community and about which respondents in the present study indicated there were differences with meat from other chicken breeds; (iii) encourage and support poultry farmers to legally register their farms and certify them as organic, as preliminary steps to facilitate the marketing of Utrerana chicken products; (iv) a study that is already being carried out during 2024 consists of a survey of the citizens of the municipality of Utrera to investigate their knowledge, perceptions, and consumption habits of Utrerana chicken products, which will be useful to propose measures and strategies to increase knowledge and dissemination of the breed among the population. It is proposed to undertake a similar study throughout Andalusia.

## 5. Conclusions

This study has shown that the maintenance and breeding of the Utrerana chicken, an egg-oriented breed native to Spain, are currently carried out by smallholders based mainly in Andalusia (southern Spain), many of them in the Seville countryside, who maintain as a complementary activity small flocks aimed at self-supply and breeding for morphological beauty. The Utrerana breed is endangered since its commercial farming was abandoned about six decades ago, the current farms being created from the 1970s onwards, and to a greater extent from the end of the 2000s. The Partridge variety is the most widespread of the breed, followed by the Black and Black-barred varieties, with the White variety being found in very small numbers. Housing and husbandry are similar to those followed at the origins of the breed in the mid-1920s and are based in poultry houses with access to outdoor enclosures, where the chickens graze, sunbathe, and feed on what they find, also providing them with grains and compound feed. The health status and welfare levels maintained on Utrerana chicken farms are good, to which the breed’s rusticity, low housing densities, and non-intensive management contribute. Due to the abandonment of selection for productive criteria, the current laying performance is low, around three-quarters of that achieved in the 1950s. The marketing of products is very limited and local, with the sale of table eggs and breeding birds predominating, and the sale of meat being very scarce, partly due to the difficulties in marketing because there are few legally registered farms and the production volume is small. For this reason, few farms carry out promotional and advertising activities. Currently, the fairs and morphological competitions organized by livestock associations in Andalusia, especially the Utrerana Chicken Fair organized by ANCGU, support the dissemination of the breed and help the economy of these farmers. In this context, the consolidation and expansion of the Utrerana chicken breed would benefit from the implementation of support programmes by the competent authorities, aimed at genetic improvement of the laying performance of the hens and at valorising their productions by facilitating marketing.

## Figures and Tables

**Table 1:** Groups of variables and questions included in the survey administered to Utrerana chicken farmers.

Groups of Variables and Questions
Year of establishment and geographic location
Farm size and flock structure
Administrative status, productive specialisation and other agricultural and livestock activities of the farm
Bird keeping facilities and equipment
Egg production and reproductive lifespan
Husbandry practices
Feeding management
Biosecurity, animal welfare and pathology
Marketing, promotion and advertising

**Table 2:** Number of roosters and hens per farm, in total and by variety of the Utrerana breed (n = 25).

Bird Type	Farms Keeping Birds ^1^		Number of Birds per Farm			
	n	%	Mean ± SE	Mininum	Maximum	Mode
Hens:						
Partridge	21	84	32.1 ± 9.97	1	150	6
Black-barred	15	60	22.5 ± 11.3	2	170	3
Black	14	56	22.1 ± 6.96	3	90	4
White	5	20	3.60 ± 1.44	1	8	1
Total Hens of any variety	25	100	53.5 ± 17.1	1	340	9
Roosters:						
Partridge	18	72	4.83 ± 1.81	1	30	1
Black-barred	10	40	3.40 ± 1.43	1	15	1
Black	13	52	3.23 ± 0.802	1	10	1
White	6	24	2.17 ± 0.980	1	7	1
Total Roosters of any variety	24	96	7.33 ± 2.43	1	55	1
Total birds of any variety:	25	100	60.6 ± 18.6	2	341	8

^1^ Number and percentage of farms that keep Utrerana chickens of each variety and sex. SE: Standard error.

**Table 3:** Administrative status and productive specializations of the Utrerana chicken farms (n = 25).

Variable	n	%
It is registered in the General Registry of Livestock Farms	10	40.0
It is registered as a zoological nucleus	0	0.000
The farm is certified organic	1	4.00
Produces meat	4	16.0
Produces eggs	18	72.0
It is a self-supply farm	18	72.0
It is a hobby farm	18	72.0
Produces breeder birds	14	56.0
Raises other chicken breeds	17	68.0
Raises other livestock species	11	44.0
Grows agricultural products	13	52.0

**Table 4:** Number and surface area of indoor poultry houses and outdoor enclosures or pens in the Utrerana chicken farms.

Variable	Farms with Facilities (n)	Mean ± SE	Mininum	Maximum	Mode
Number of poultry houses	25	2.92 ± 0.583	1	12	1
Surface area of poultry houses (m^2^)	25	179 ± 81.18	6	2000	30
Number of outdoor enclosures or pens	24	1.72 ± 0.358	0	9	1
Surface area of outdoor enclosures or pens (m^2^)	24	3381 ± 837	10	12,000	1000

SE: Standard error.

**Table 5:** Bird stocking density in the poultry house, surface available per bird in the enclosure, and bird density per feeder and drinking trough in the Utrerana chicken farms (n = 25).

Variable	n	%
Stocking density ^1^:		
The number of laying hens in the poultry house is less than 6/m^2^	17	68.0
The stocking density of fattening chickens in the poultry house is less than 21 kg BW/m^2^	15	60.0
The surface area per bird in outdoor enclosures or pens is greater than 4 m^2^	23	92.0
Bird density per feeder and drinking trough ^2^:		
It has automatic feeders	3	12.0
It has linear feeders with more than 10 cm/bird	14	56.0
It has hopper feeders with more than 4 cm/bird	19	76.0
It has automatic drinking troughs	13	52.0
It has channel drinking troughs with more than 2.5 cm/bird	13	52.0
It has circular drinking troughs with more than 1 cm/bird	5	20.0
Have at least one nipple or cup drinking troughs for every 10 birds	7	28.0

^1^ According to the Commission Implementing Regulation (EU) 2020/464 [42]. ^2^ According to the Royal Decree 3/2002 [43]. BW: Body weight.

**Table 6:** Egg production and reproductive lifespan in the Utrerana chicken farms (n = 25).

Variable	Mean ± SE	Mininum	Maximum	Mode
Egg production:				
Egg production per hen per year (n)	139 ± 10.78	20	210	100
Annual egg production per farm (n)	4996 ± 1773	200	40,800	2000
Reproductive lifespan:				
Reproductive lifespan (months)	49.1 ± 3.73	12	96	60
Culling rate (%)	15.4 ± 2.43	5	60	10

**Table 7:** Husbandry practices in the Utrerana chicken farms (n = 25).

Variable	n	%
Composts bird manure	8	32.0
Uses artificial photoperiod supplementation to stimulate reproduction	1	4.00
Incubate eggs	17	68.0
Carries out artificial incubation	10	40.0
Carries out natural incubation	12	48.0
Slaughters fattening chickens with at least 81 days of age	17	68.0

**Table 8:** Feeding management in the Utrerana chicken farms (n = 25).

Variable	n	%
Grows vegetables for bird feeding	15	60.0
Birds have access to trees	13	52.0
Uses compound feed	10	40.0
The farmer knows whether the compound feed used includes ingredients with genetically modified organisms	1	4.00
Prepares its own feeds	12	48.0
Uses vitamins and minerals	10	40.0

**Table 9:** Biosecurity, animal welfare, and pathology in the Utrerana chicken farms (n = 25).

Variable	n	%
Biosecurity and prophylaxis measurements:		
Ventilation of poultry houses is adequate	24	96.0
Controls rodents	24	96.0
Disinfects feeders and drinking troughs	24	96.0
Removes dead birds	24	96.0
Removes the litter	22	88.0
Vaccinates birds	7	28.0
Incidence of diseases:		
Intestinal parasites	3	12.0
Mites	6	24.0
Diarrhoea	0	0.000
Respiratory diseases	5	20.0
Welfare issues:		
Beak trimming	0	0.000
Plucks live birds	0	0.000
Belongs to a Livestock Health Defence Group	1	4.00

**Table 10:** Marketing, promotion, and advertising activities of the Utrerana chicken farms (n = 25).

Variable	n	%
Associative life and participation in livestock fairs:		
The farmer is affiliated with the ANCGU	17	68.0
Participates in fairs and conformation shows	11	44.0
Sells live birds at fairs	7	28.0
Their chickens have been awarded in conformation shows	9	36.0
Receives subsidies for raising Utrerana chickens	1	4.00
Promotional and advertising activities:		
Has an own website	2	8.00
Uses social networks	6	24.0
Advertise their products in the press	1	4.00
Publishes magazine articles about the Utrerana chicken	1	4.00
Marketing:		
Sells hatching eggs	11	44.0
Sells table eggs	18	72.0
Sells meat	4	16.0
Market area reached:		
Local	10	40.0
Regional	2	8.00
Nationwide	1	4.00
Exports	0	0.000

ANCGU: Asociación Nacional de Criadores de Gallinas Utreranas (National Association of Utrerana Chicken Breeders).

## Data Availability

The data presented in this study are available on request from the corresponding author to avoid compromising the anonymity of the participants.
